# Generation and analysis of ESTs from strawberry (*Fragaria xananassa*) fruits and evaluation of their utility in genetic and molecular studies

**DOI:** 10.1186/1471-2164-11-503

**Published:** 2010-09-17

**Authors:** Aureliano Bombarely, Catharina Merchante, Fabiana Csukasi, Eduardo Cruz-Rus, José L Caballero, Nieves Medina-Escobar, Rosario Blanco-Portales, Miguel A Botella, Juan Muñoz-Blanco, José F Sánchez-Sevilla, Victoriano Valpuesta

**Affiliations:** 1 Departamento de Biología Molecular y Bioquímica. Universidad de Málaga. Spain; 2 Departamento de Farmacognosia. Universidad de Córdoba. Spain; 3 IFAPA Centro Churriana. Málaga. Spain

## Abstract

**Background:**

Cultivated strawberry is a hybrid octoploid species (*Fragaria xananassa *Duchesne ex. Rozier) whose fruit is highly appreciated due to its organoleptic properties and health benefits. Despite recent studies on the control of its growth and ripening processes, information about the role played by different hormones on these processes remains elusive. Further advancement of this knowledge is hampered by the limited sequence information on genes from this species, despite the abundant information available on genes from the wild diploid relative *Fragaria vesca*. However, the diploid species, or one ancestor, only partially contributes to the genome of the cultivated octoploid. We have produced a collection of expressed sequence tags (ESTs) from different cDNA libraries prepared from different fruit parts and developmental stages. The collection has been analysed and the sequence information used to explore the involvement of different hormones in fruit developmental processes, and for the comparison of transcripts in the receptacle of ripe fruits of diploid and octoploid species. The study is particularly important since the commercial fruit is indeed an enlarged flower receptacle with the true fruits, the achenes, on the surface and connected through a network of vascular vessels to the central pith.

**Results:**

We have sequenced over 4,500 ESTs from *Fragaria xananassa*, thus doubling the number of ESTs available in the GenBank of this species. We then assembled this information together with that available from *F. xananassa *resulting a total of 7,096 unigenes. The identification of SSRs and SNPs in many of the ESTs allowed their conversion into functional molecular markers. The availability of libraries prepared from green growing fruits has allowed the cloning of cDNAs encoding for genes of auxin, ethylene and brassinosteroid signalling processes, followed by expression studies in selected fruit parts and developmental stages. In addition, the sequence information generated in the project, jointly with previous information on sequences from both *F. xananassa *and *F. vesca*, has allowed designing an oligo-based microarray that has been used to compare the transcriptome of the ripe receptacle of the diploid and octoploid species. Comparison of the transcriptomes, grouping the genes by biological processes, points to differences being quantitative rather than qualitative.

**Conclusions:**

The present study generates essential knowledge and molecular tools that will be useful in improving investigations at the molecular level in cultivated strawberry (*F. xananassa*). This knowledge is likely to provide useful resources in the ongoing breeding programs. The sequence information has already allowed the development of molecular markers that have been applied to germplasm characterization and could be eventually used in QTL analysis. Massive transcription analysis can be of utility to target specific genes to be further studied, by their involvement in the different plant developmental processes.

## Background

Strawberry (*Fragaria xananassa *Duchesne ex. Rozier) is one of the most important berry crops in the world; in 2008 its production was approximately 4 million metric tons [1]. The benefits that strawberry fruit consumption has on cardiovascular, neurodegenerative, and other diseases like aging, obesity, and cancer has been a subject of increased study over recent years [2]. The strawberry belongs to the family Rosaceae in the genus *Fragaria*. There are four basic fertility groups in *Fragaria *that are associated primarily with their ploidy level or chromosome number. The most common native species, *F. vesca *L., has 14 chromosomes and it is considered to be a diploid and proposed as model for the genus [3]. The most important cultivated strawberry is a perennial and herbaceous octoploid plant, with fifty six chromosomes (2n = 8× = 56), that stems from the cross of the octoploids *F. virginiana *Duchesne from eastern North America, which was noted for its fine flavour, and *F. chiloensis *(L.) Mill. from Chile, noted for its large size [3]. Numerous varieties of strawberries have been developed in the temperate zones of the world by different breeding programs.

Strawberry has been considered a non-climacteric fruit, since there is no concomitant burst of respiration and production of the hormone ethylene that triggers the ripening process [4,5]. The berry results from the development of the flower receptacle that consists of a pith at the centre, a fleshy cortex, an epidermis, and a ring of vascular bundles with branches leading to the achenes, the true fruits. Each achene contains a single seed and a hard pericarp. The achenes are attached to the receptacle by vascular strands. When classifying the strawberry as non-climacteric, no distinction was made between the receptacle and the achenes. Growth and ripening of strawberry fruits is an important field of research, which includes the role played by hormones, the synthesis of anthocyanins and flavour compounds, and the cell wall changes occurring during the late stages of ripening. It is reasonable to think that those changes that are important for fruit quality, like anthocyanins and flavour content, as well as fruit softening, mostly rest on the receptacle, whereas hormone control of the process might be supported by the achenes. Therefore, the generation of tools to distinguish the functional roles of these two parts in the growth and ripening of the whole berry is important.

The hormone auxin, which is supplied by the achenes, is considered as a key regulator of growth and ripening. Removal the achenes from the receptacle has different effects depending on the developmental stage. In the early green stage it stops receptacle growth, whereas in the late green and white stages it accelerates ripening [6]. Interestingly, both effects are suppressed by the exogenous application of auxin restoring normal development [7], [8]. Therefore the role of ethylene in fruit ripening has been considered as negligible. Recently, however, it has been reported that the achenes of red fruits produce ethylene at low concentrations, although its role in fruit ripening is unclear [5].

Genes related to biochemical processes and metabolites, such as the health promoting metabolites anthocyanin [9] and vitamin C [10], with important roles in modulating fruit quality have been studied. The aroma, an important criterion defining strawberry quality is dependent on more than 360 volatile compounds, many of them esters, whose synthesis is dependent on the strawberry alcohol acyltransferase (SAAT) activity encoded by the FaSAAT gene [11]. Of all the volatiles, furaneol (HDMF) is the main one responsible of the aroma of the strawberry fruit [12]. The genes of two enzymes related to the biosynthesis of HDMF have been cloned [13], [14]. Due to the importance of the cell wall in the integrity of the strawberry fruit, genes encoding for cell wall modifying enzymes have been analysed, including expansins [15], cellulases [16], beta-galactosidase [17], pectate lyases [18], [19], and pectinmethylesterases [20], [21].

Despite all the previous molecular studies, including a recent report on metabolic changes during fruit growth and ripening [22], information on regulatory genes involved in the strawberry fruit development is still scarce. The development of genomic tools will, no doubt, constitute important input that will facilitate strawberry research. In recent years molecular markers for this species have been developed [23], [24], and microarray gene expression experiments during fruit ripening [25], [26], and in relation to fruit firmness have been reported [27].

One of the most useful tools in the gene discovery, and further assignment of function, is the availability of expressed sequence tags (ESTs). These sequences stem from cDNA libraries constructed from different tissues and organs, under different environmental conditions and stages of development, so they represent a broad set of expressed genes. ESTs collections have been used in gene expression studies [28] and to saturate genetic maps with simple sequence repeats (EST-SSRs) [29] or single nucleotide polymorphisms (SNPs) [30]. They also allow the identification of miRNA precursors and targets [31], and massive transcriptome analysis using microarrays [32], [33]. At present there are more than 50 million ESTs in the GenBank database, a quarter of which are from plants. Although fruit crops have been less studied than other plants like Arabidopsis, rice, soybean, maize or pine, there is a significant number of ESTs obtained from fruits like tomato [34], grape [35], apple [36], citrus [37] and melon [38].

In this report we have analysed around 10,000 ESTs from *F. xananassa*, 4,600 of which originated from our own sequencing project, and 5,400 are from the GenBank database. These ESTs have been processed, clustered, annotated and classified into different functional categories. We have searched for SSRs and SNPs in the ESTs set in order to evaluate their potential in marker-assisted breeding programs. Creation of a gene index [39] and comparisons with other species enabled the conclusion that the highest average sequence identity was with the wild diploid relative *F. vesca*, up to a value of 93.27% between sequences of orthologous genes. Expression studies of selected ESTs using QRT-PCR allowed investigating on the possible involvement of hormones like auxin, ethylene, and brassinosteroid in strawberry fruit ripening. In addition, the set of non-redundant sequences from *F. xananassa *jointly with an equivalent number of sequences from *F. vesca *has been used to design and perform a microarrays-based expression studies in ripe receptacle of these two species.

## Results

### EST Sequencing and Clustering

More than 4,500 clones were sequenced from six cDNA libraries prepared from fruits of several varieties of the cultivated strawberry (*F. xananassa*) (Table 1). Because we are interested in fruit ripening, transcripts were extracted from two ripening stages, green and red, and two different fruit parts: achenes and receptacle. In addition, transcripts from red fruits were favoured vs. transcripts from green fruits using two different subtraction procedures (see Methods). Also, sequences were obtained from transcripts corresponding to genes differentially expressed in ethylene-treated ripe fruits. A web-accessible database containing all the EST sequences, contigs, and bioinformatic tools for their analysis and data mining has been created and named FREST http://fresa.uco.uma.es/srs71. The set was completed with the dbEST GenBank sequences of *F. xananassa*. In total, 10,018 sequences were analyzed in the present study (Table 2).

**Table 1:** Description on cDNA libraries

Library	Cultivar	Tissue/stage/treatment	Source
M1	Carisma	Green fruit receptacle	This publication
M2	Carisma	Green fruit achenes	This publication
C1	Chandler	Subtracted red/green fruit	This publication
C2	Chandler	Red fruit	This publication
C3	Chandler	Subtracted red/green fruit	This publication
L1	Elsanta	Red fruit treated with ethylene	This publication
CO3	Queen Elisa	Red fruit	dbEST GenBank
CO8	Festival	Whole plant treated 24 h with 1 mM SA	dbEST GenBank
AB2*	Shikinari	Mature leaf	dbEST GenBank
AF0*	Brighton	Red fruit receptacle	dbEST GenBank
AI7*	Elsanta	Red fruit	dbEST GenBank
CO7*	Pajaro	Leaves infected with *Colletotrichum*	dbEST GenBank

* These libraries (with less than 100 sequences) were pooled for their analysis and named here after as SGBL (small GenBank libraries)

**Table 2:** ESTs information and clustering.

Library	Raw sequences	Good-quality ESTs	EST length	bp/N average	Singletons	Contigs (Average size)	Unigene (Average size)	
M1	1908	1863	534±98	155	1069			
M2	1265	1239	537 ± 107	139	760			
C1	358	350	549 ± 179	277	156			
C2	403	398	609 ± 186	170	226	1120	7096	
C3	358	355	484 ± 155	316	163			
L1	350	343	343 ± 68	291	109	(619.74 ± 195.83)	(491.25 ± 195.83)	
CO3	3753	3644	359 ± 175	51	2465			
CO8	1511	1497	612 ± 150	548	974			
SGBL	112	101	385 ± 158	398	54			
Total	10018	9790			5976			

Clustering and determination of the consensus sequences were performed through the TGICL pipeline as described in the Methods section.

The raw sequences were processed to remove vector and adaptor sequences and to discard sequences with either more than 3% of N or being less than 100 bp in length. The mean length ranged from 343 to 612 bp, and the accuracy was evaluated by the frequency of appearance of an undetermined nucleotide, and changed from an average of once every 51 to 548 bp (Table 2). All ESTs (9,790) that passed the quality control were used for clustering. A total of 5,976 singletons and 1,120 contigs/tentative consensuses were obtained, resulting in 7,096 unigenes/non redundant sequences (Table 2). Some genes were represented by multiple ESTs as shown in the Table 3 that includes the contigs with more than 15 ESTs. In the case of contigs corresponding to metallothionein-like and prunin, with more than 100 ESTs for each one, it is notable that they are overrepresented in the M1 (green receptacle) and M2 (green achenes) libraries, respectively (Table 3). This is related to the high expression level of these genes in these fruit parts, i.e. the receptacle and achenes, at this developmental stage.

**Table 3:** Contigs made up of more than 15 ESTs

Contig	Number of ESTs	Number of libraries	Annotation	M1 library abundancy (‰)	M2 library abundancy (‰)
CL0001Cg02R	121	7	Metallothionein-like Protein related cluster	22.01	4.29
CL0002Cg01R	51	4	Pru2 proteinprecursor related cluster	0.54	24.69
CL0007Cg01R	34	2	Pru2 Protein precursor related cluster	0.54	17.71
CL0002Cg04R	32	2	Pru2 protein precursor related cluster	2.68	14.49
CL0004Cg03R	24	2	Putative oxidoreductase related cluster	0	0
CL0009Cg01R	22	4	Ethylene-forming- enzyme-like dioxygenase	0	0
CL0002Cg02R	21	3	Prunin precursor related cluster	1.61	9.13
CL0006Cg02R	21	5	Metallothionein-like protein type 2 MET1	5.90	2.15
CL0016Cg01R	19	6	Putative aldo/keto reductase related cluster	1.07	0
CL0001Cg06R	18	1	No significant similarity found	0	0
CL0003Cg03R	18	4	HyPRP related cluster	2.68	0
CL0019Cg01R	18	6	Calmodulin 2/3/5 related cluster	1.07	1.07
CL0011Cg01R	17	4	Translationally controlled tumor protein	2.68	0
CL0010Cg01R	16	4	Lipid transfer protein Precursor	3.76	0
CL0023Cg01R	16	2	G protein-coupled receptor-like protein	8.05	0
CL0026Cg01R	16	5	Plasma membrane intrinsic protein	3.22	0
CL0025Cg01R	15	2	Putative 70 kDa peptidylprolyl isomerase	7.51	0
CL0005Cg03R	15	4	Arabidopsis low temperature and salt responsive protein	1.07	0

### Functional annotation

A summary of the different parameters studied in the annotation of the complete set of unigenes from *F. xananassa *is shown in Table 4. The number of chimeras was very low based on the BlastX sequence searches against the Arabidopsis TAIR Database. Annotation included not only sequence homology comparisons in the GenBank at two e-value cutoff (47.7% of sequences at e-value < 1e-10, and 1.5% at e-value < 1e-100), but also search for domains using InterProScan (9.2% of sequences), signal peptides using signalP tool (17.5% of sequences), association to gene ontology (GO) terms (29.9% of sequences), and numbers of the Enzyme Commission (EC) (7.2% of sequences). In total, a 56.1% unigenes were annotated in at least one of the categories of Table 4. This means that there is still a 43.9% of the unigenes that remained unknown for any putative function.

**Table 4:** Annotation of strawberry unigenes

Annotation Tool	*Fragaria xananassa* dataset
Chimera analysis using TAIR blastX	8 (0.1%)
GenBank blastX (e-value < 1e-10)	3454 (48.7%)
GenBank blastX (e-value < 1e-100)	109 (1.5%)
InterProScan (Domain Databases)	653 (9.2%)
InterProScan (SignalP)	1244 (17.5%)
GO Terms associated	2120 (29.9%)
EC number associated	512 (7.2%)
Unigenes with annotation	3983 (56.1%)

**Figure 1:**
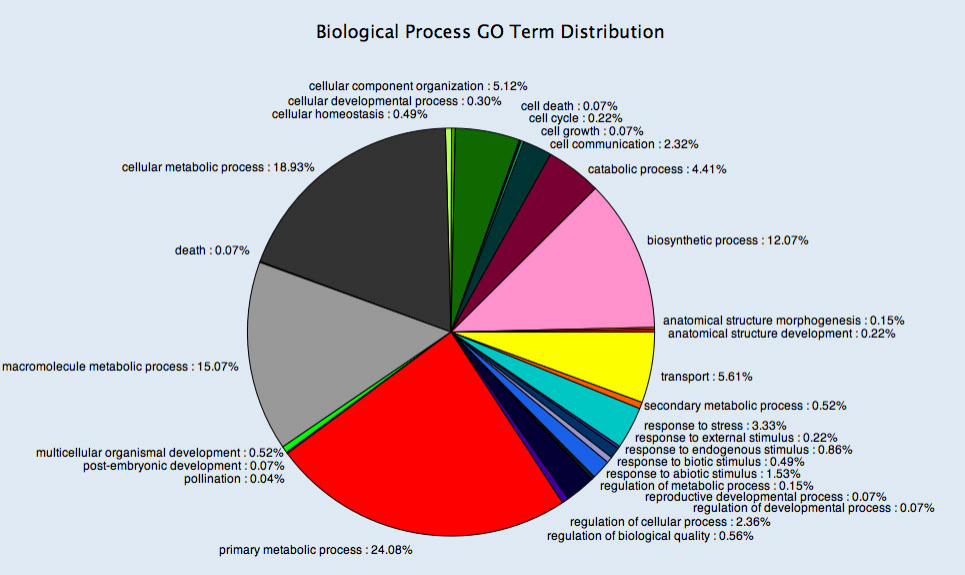
**Distribution of *F. xananassa *unigenes with associated GO terms by biological processes**. EST sequences from strawberry with assigned GO terms according to the Blast2GO software were grouped, at the level 3, by Biological Processes involved.

Further, a global analysis by gene ontology (GO) groups was performed with the Blast2GO software [40]. Blast2GO uses different tools as BlastX and InterProScan to annotate sequences. Figure 1 shows the result of this analysis. Metabolic processes account for almost 60% of the annotated sequences, including primary, macromolecule, and cellular metabolic processes. Remarkable is the dominance of biosynthetic processes (12.07%) over catabolic processes (4.41%). Proteins involved in transport are represented by 5.61%, and other groups of proteins encoded by EST correspond to a wide variety of biological processes.

### Sequence analyses allowed the identification of genes involved in metabolic and regulatory processes of fruit ripening

We were interested in the fruit ripening process; therefore the RNA used in the preparation of the cDNA libraries of the present publication was extracted from two parts of the berry at two developmental stages, as it was ripening-enriched by subtraction (Table 1). There has been previous sequencing project in strawberry focused on the fruit ripening [25] and we now complete this previous information. We have performed manual assignment of *F. xananassa *unigenes to specific metabolic and signalling pathways (Additional files 1, 2, 3 and 4) providing an exhaustive catalogue of *F. xananassa *sequences to be further used in specific research projects. We summarize in Table 5 the contribution of the new sequences to the information previously available on strawberry genes relevant for the fruit ripening process. In 8 of the 21 metabolic pathways the new genes account for more than 50 percent of the total number of genes known. More interestingly, in hormone signalling the information on new genes is very significant, being over 50 percent in 5 of the 6 pathways. In some cases, like brassinosteroid, gibberellins and abscisic acid, there is not previous information on gene sequences of the corresponding signalling pathways.

**Table 5:** Genes involved in metabolic and regulatory processes relevant for the ripening of the strawberry fruit

Metabolic Pathway	Novel ESTs	ESTs described previously	Novel genes	Genes described previously	Novelty M**(%)**
Glycolysis	38	22	3	6	33
TCA Cycle	14	5	1	3	25
Pentose Phosphate	7	2	4	2	67
Pathway					
Fatty Acid Biosynthesis	23	6	5	2	71
Aminoacid Biosynthesis	28	34	5	12	29
Urea Cycle	5	0	4	0	100
Sucrose Metabolism	8	1	2	1	67
Starch Metabolism	16	7	2	3	40
Sorbitol Metabolism	0	1	0	1	0
Isoprenoid Pathway	10	3	1	3	25
Terpenoid Biosynthesis	5	2	0	1	0
Sterol Biosynthesis	1	2	0	2	0
Carotenoid Biosynthesis	1	0	1	0	100
Phenylpropanoid Pathway	12	21	3	4	43
Lignin Biosynthesis	23	11	2	4	33
Flavonoid Biosynthesis	22	14	4	5	44
Brassinosteroid	1	0	1	0	100
Biosynthesis					
Gibberellin Biosynthesis	3	0	2	0	100
Abscisic Acid	0	1	0	1	0
Biosynthesis					
Ethylene Biosynthesis	6	9	0	2	0
Jasmonic Acid	3	1	3	1	75
Biosynthesis					

**Hormone Signaling**	**Novel ESTs**	**ESTs described** **previously**	**Novel genes**	**Genes described** **previously**	**Novelty** **(%)**

Auxin	16	11	8	6	57
Brassinosteroid	6	0	3	0	100
Cytokinin	1	2	0	2	0
Gibberellin	4	0	2	0	100
Abscisic Acid	1	0	1	0	100
Ethylene	10	5	3	2	60

### Overall comparison of *F. xananassa *sequences with other species. Gene Index

We determined the *F. xananassa *gene index and related it to different plant species using the DFCI Gene Index Database (for species like Arabidopsis thaliana, *Oryza sativa *or *Vitis vinifera*), and "ad hoc" gene indices created from the GenBank dbEST (for species like *Prunus persica*, *Prunus armeniaca*, *Citrus *spp. or *Fragaria vesca*). The homology search was performed using the BlastN tool against non-redundant sequences and true orthologues were considered as having E values of ≤ 1e-20. Results of this analysis are shown in Table 6. The analysis of these orthologous groups was made from three different perspectives. Percentage of orthologous unigenes of each species relative to *F. xananassa *unigenes, percentage of *F. xananassa *orthologous unigenes relative to each species unigenes, and the average identity, after the alignments, of unigenes from each species with the corresponding *F. xananassa *orthologous unigene. Values for the first two comparisons (Table 6, columns 2,3) are highly dependent on both the number and the length of the available sequences for the species compared. We focus on the values obtained for *F. vesca*, the wild diploid species of the same genus of *F. xananassa*. It is noteworthy that there is a 36.22 percent of unigenes from cultivated strawberry that show no putative orthologues to *F. vesca *unigenes (Table 6, column 3). Thus, although the number of ESTs available for *F. vesca *is more than four-fold the number of ESTs for *F. xananassa *there are a high number of sequences of cultivated strawberry that have not been revealed in the *F. vesca *sequencing projects.

**Table 6:** Global homology comparison between sequences of different species

Species	Unigenes of X species with high homology to strawberry (%)	Unigenes of strawberry with high homology to X species (%)	Average identity for the alignments (%)
*Fragaria vesca*	32.42	63.78	93.27
*Rosa hybrid*	71.94	15.66	91.04
*Prunus dulcis*	62.67	8.16	87.53
*Prunus armeniaca*	54.25	23.00	87.46
*Prunus persica*	30.33	37.92	87.43
*Malus x domestica*	55.36	46.53	86.81
*Theobroma cacao*	37.65	7.31	85.04
*Citrus clementina*	30.75	15.26	84.86
*Vitis vinifera*	19.27	26.18	84.77
*Glycine max*	11.97	26.14	84.66
*Cucumis sativus*	32.21	8.10	84.65
*Citrus sinensis*	26.42	24.79	84.63
*Triticum aestivum*	15.81	10.88	84.59
*Hordeum vulgare*	22.23	10.53	84.52
*Cucumis melo*	32.48	8.10	84.51
*Mesembryanthemum crystallinum*	23.97	11.99	84.51
*Oryza sativa*	5.44	11.47	84.39
*Saccharum officinarum*	8.49	10.70	84.33
*Zea mays*	6.60	10.77	84.27
*Medicago truncatula*	11.19	21.70	84.23
*Nicotiana benthamiana*	19.84	9.01	84.22
*Gossypium*	16.49	24.90	84.22
*Capsicum annuum*	17.81	12.34	84.17
*Solanum lycopersicum*	12.46	17.15	84.15
*Solanum tuberosum*	11.25	17.50	84.12
*Brassica napus*	19.33	11.49	84.09
*Arabidopsis thaliana*	7.60	15.57	83.87
*Pinus*	8.71	7.69	83.78
*Ananas comusus*	21.81	5.21	83.68

The average identity was calculated after the alignments of these putative orthologous sequences from different species with the *F. xananassa *sequences (Table 6, column 4). As expected, the highest value was for *F*. vesca reaching the 93.27 percent, as the genome of this species probably shares a common ancestor with *F. xananassa *[42]. The order of the species in this column reflects the taxonomic proximity with close relatives, having *Rosa hybrid*, *Prunus *and *Malus *the highest values. However, this is not an analysis of phylogeny, but the result of the multiple alignments of sequences available in the databases for the different species. Therefore, it is not possible to gain taxonomic information from the results here presented on species out of the Rosaceae family (Table 6, column 4)

### Actual polymorphisms evaluation inside the EST collection

Microsatellites, or simple sequence repeats (SSRs), are stretches of DNA consisting of tandem repeated short units of 1-6 base pairs in length. The uniqueness and the value of microsatellites as molecular markers arise from their multiallelic nature, co dominant inheritance, relative abundance, extensive genome coverage and simple detection by PCR. Three hundred eighty three (4.64%) SSRs were identified in 329 of the 7.096 unigenes. Fifty sequences contained more than 1 SSR and 47 of them with less than 100 bp between 2 consecutive SSRs. The frequency of SSR was one every 9.1 kb of the sequence. As shown in Table 7, dinucleotides are the most frequent motifs (47.3%), followed by trinucleotides (45.9%). Other nucleotide combinations are poorly represented (3.9% tetranucleotides, 2.9% pentanucleotides). Most of the SSRs found were on the 5' non-coding regions upstream of putative ORFs, close to the initial ATG. A total of 102 SSRs have been amplified and 10 have already been used for studies of *F. xananassa *varieties and *Fragaria *species [24].

**Table 7:** Simple sequence repeats (SSRs) statistics

Dinucleotide repeat	Number of di-pSSR	%
AC/GT	6	3.3
AG/CT	136	75.1
AT/AT	39	21.6
**Total**	**181**	**100**

**Trinucleotide repeat**	**Number of Tri-pSSR**	**%**

AAC/GTT	9	5.1
AAG/CTT	59	33.5
AAT/ATT	3	1.7
ACC/GGT	21	11.9
ACG/CTG	9	5.1
ACT/ATG	25	14.2
AGC/CGT	15	8.5
AGG/CCT	18	10.2
AGT/ATC	11	6.3
CCG/CGG	6	3.4
**Total**	**176**	**100**

**Tetranucleotide repeat**	**Number of tetra-pSSR**	**%**

AAAC/GTTT	1	6.7
AAAG/CTTT	5	33.3
AAAT/ATTT	6	40
ACGT/ATGC	1	6.7
ACTC/AGTG	1	6.7
AGCT/ATCG	1	6.7
**Total**	**15**	**100**

**Tetranucleotide repeat**	**Number of tetra-pSSR**	**%**

AAAAC/GTTTT	1	14.3
AAAAG/CTTTT	1	14.3
AAAGC/CGTTT	1	14.3
AAGAG/CTCTT	3	42.8
AATGT/ACATT	1	14.3
**Total**	**7**	**100**

The number of di-, tri-, tetra and pentanucleotides repeats identified in the strawberry database is shown for the complete set of putative SSRs (pSSRs)

Of the 1,120 contigs generated in the present study, 242 contained a minimum of two alleles, 128 of them with potential SNPs. In these contigs the changes corresponded to 636 potential SNPs and 148 indels. The final number of good quality true-SNPs was 372, 192 of them were transitions, 124 were transversions, 2 were tri-allelic polymorphisms, and 54 were indels. The frequency of SNP was one every 256 bp, and a mean value of 2.9 SNPs per contig.

### Expression analysis of selected genes during fruit ripening

**Figure 2:**
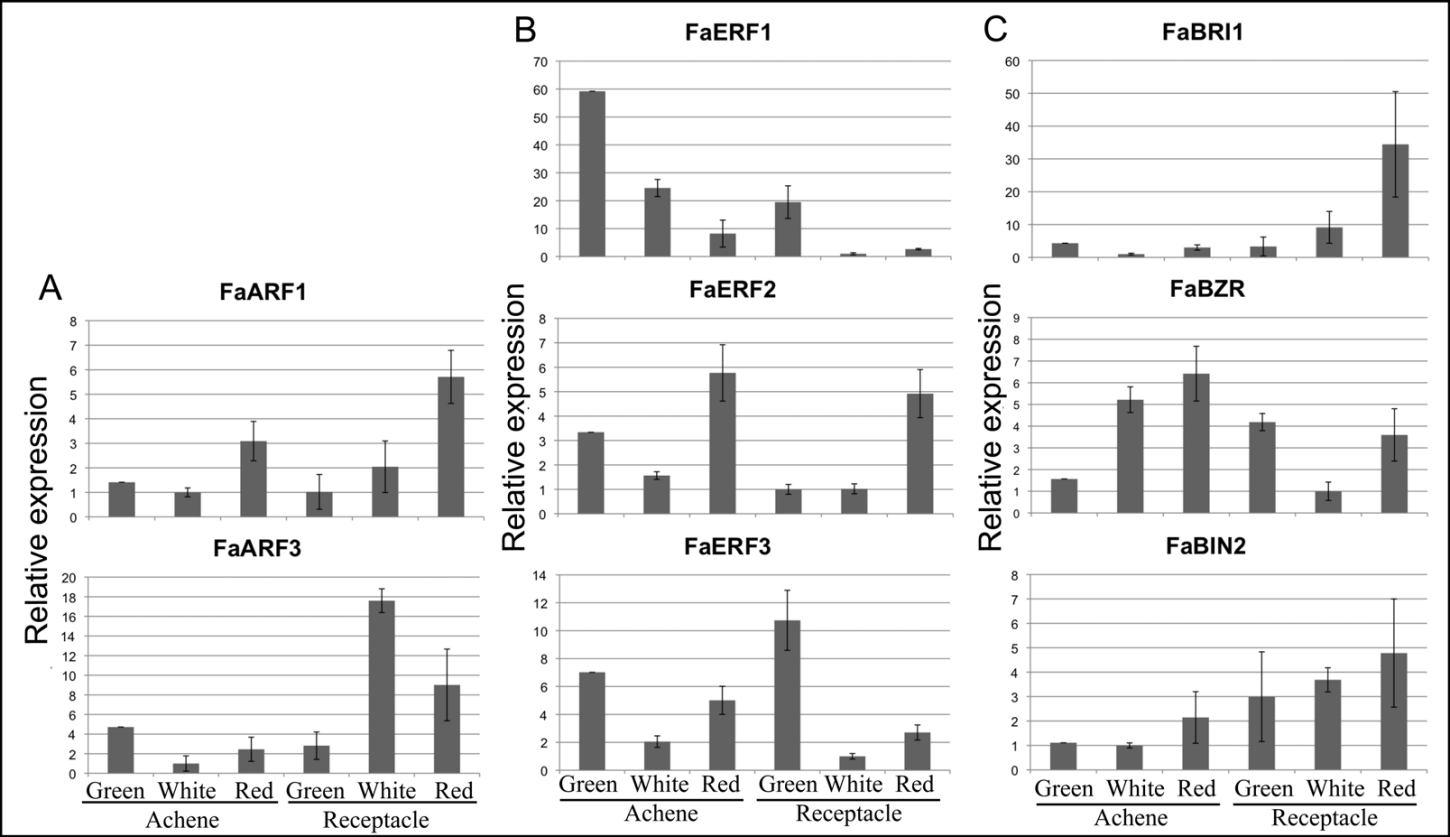
**Relative expression of ARF, ERF, brassinosteroid signalling pathway genes from strawberry in achenes and receptacle of fruits at three developmental stages, evaluated by QRT-PCR**. RNA was extracted separately from achenes and receptacle of strawberry fruits at three developmental stages corresponding to green, white and red receptacle, as previously described [79]. Real time quantitative PCR was performed as described in the Methods sections. The values are the results of two biological and three technical repetitions ± standard error.

A detailed catalogue of strawberry sequences of genes related to hormone biosynthesis and signalling pathways is shown in Table 8. The expression of some of the hormone-related genes whose sequence information is provided in the present paper (Table 9) was further studied in fruits. For auxin we selected genes encoding ARF (auxin response factor) proteins, which are transcription factors controlling the expression of auxin-induced genes [43]. Regarding ethylene, we studied genes encoding ethylene response transcription factors (ERF) that belong to the large AP2/ERF family regulating ethylene-responsive genes [44]. Patterns of the expression of these genes in achenes and receptacle at three developmental stages are shown in Figure 2(A, B). Values of expression by qRT-PCR are relative for each gene, therefore is not possible to have information on absolute values of expression of different genes. However, it is apparent that each gene presents a tissue- and developmental-specific expression pattern with significant differences among samples (Figure 2). Thus, transcriptional activity of *FaARF1 *is highest in red receptacle whereas for *FaARF2 *the highest level of transcripts is detected in white receptacle. For the *FaERF *genes high and significant changes occur for *FaERF1 *and *FaERF3*, having the first the highest value in green achenes and the second in green receptacle. A more conspicuous case is brassinosteroids, whose involvement in fruit developmental processes has been studied in only a few species [45], [46]. We have identified ESTs homologous to the receptor and two components of the signalling pathway (*FaBRI1*, *FaBRZ1*, *FaBIN2*) (Table 9). Their expression also varies with the fruit part, achene or receptacle, and the developmental stage (Figure 2C). Highest changes occur for the receptor *FaBRI1 *whose expression is higher in receptacle and clearly increases with ripening

**Table 8:** Sequences of genes of hormone biosynthesis and signalling

Function	Gene (or gene family)	EST identified
ABA biosynthesis	NCDE	CO818085
Brassinosteroid biosynthesis	BR6ox	GT149949
Ethylene biosynthesis	SAMS	AI795146, CO381892, CO381923, CO817276, CO817815, GT150129, CO818125, GT151766, CO816980; GT151867, GT150239, GT150959
	ACO	CO817481, CO817779, GT150063
Gibberellins biosynthesis	CPS	GT151845
	GA20ox	GT151292, GW402870
Auxin signalling	ABP	CO817279, CO817899
	IAA	CO817274, GT150547 GW402778
	ARF	GW402649, GT149793 GT148932, GT151848, GT149872
Brassinosteroid signalling	BRI1	GT149043, GT149540
	BKI1	GT150457
	BZR2/BES1	GT149361, GT149240
	BIN2	CO381946, GT150424
Cytokinin signalling	AHP	CO818010
	ARR	CO816816, GT151563
Ethylene signalling	ETR	GW402489
	ERF	GW402737, CO817183, CO817782, CO817196, CO817932, GT150183, GT150854, CO816657, GT151564
Gibberellins signalling	GID1	GW403056, GT150354
	GID2/SLY1	GT150354, M119F11R

**Table 9:** Selected ESTs from hormones signalling pathways

Hormone	Gene	EST (Acc. No.)	TBLASTX			
			
			NCBI		TAIR	
			
			Best hit acc. No. (E value)	Species	Best hit gene (E value)	Annotation
Auxin	FaARF1	M101B08R (GT148932)	BT013711 (6e-90)	L. esculentum	AT1G59750 (2e-73)	AUXIN RESPONSE FACTOR 1
	FaARF3	M112C05R (GT149872)	DQ340254 (5e-81)F	L. esculentum	AT2G33860 (2e-79)	AUXIN RESPONSE TRANSCRIPTION FACTOR 3
Ethylene	FaERF1	M202A10R (GT150854)	EX674305 (2e-68)	F. vesca	AT2G47520 (1e-26)	ERF SUBFAMILY B-2 OF ERF/AP2
	FaERF2	M208H04R (GT151407)	AM289176 (2e-45)	P. persica	AT4G17490 (2e-26)	ERF SUBFAMILY B-3 OF ERF/AP2
	FaERF3	M210G07R (GT151564)	EX676973 (3e-90)	F. vesca	AT3G16770 (3e-29)	ETHYLENE RESPONSE FACTOR 72
Brassinosteroids	FaBRI1	M102D05R (GT149043)	EX671818 (3e-96)	F. vesca	AT4G39400 (1e-32)	BRASSINOSTEROID INSENSITIVE 1
	FaBZR1	M104F08R (GT149240)	AM722051 (1e-46)	C. melo	AT1G75080 (1e-39)	BRASSINAZOLE- RESISTANT 1
	FaBIN2	M118H01R (GT150424)	AB113574 (4e-45)	L. japonicus	AT3G05840 (2e-44)	SHAGGY-LIKE KINASE

### Transcript analysis in red receptacle of *F. xananassa *and *F. vesca*

**Figure 3:**
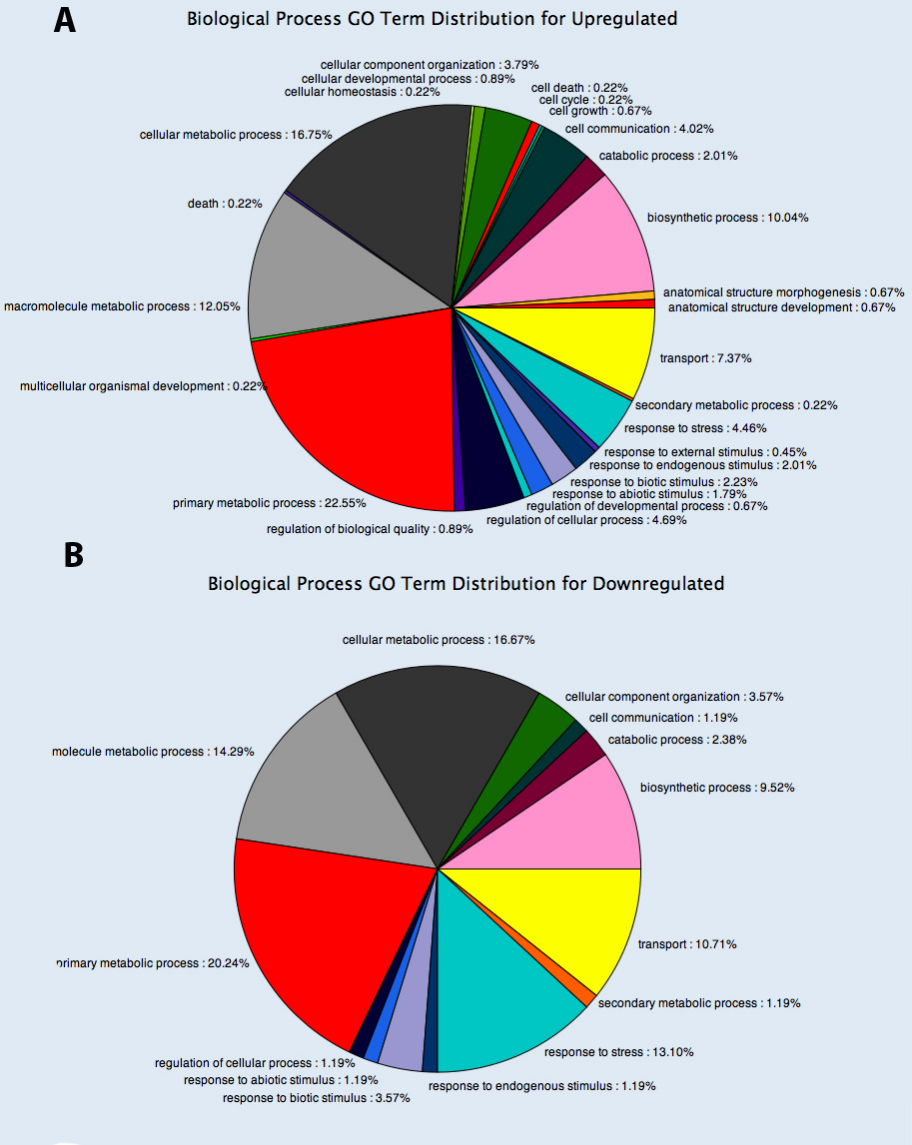
**Distribution by biological processes involved of differentially expressed genes in the ripe receptacles of *F. xananassa *compared to *F. vesca***. A) Distribution by biological processes of genes with higher expression in the ripe receptacle of *F. xananassa*. B) Distribution by biological processes of genes with lower expression in the ripe receptacle of *F. xananassa*. The analysis was performed in genes that were differentially expressed over 4-fold, and passed the t-test and FDR (Benjamini-Hochberg) for multiple testing corrections with a confidence p-value < 0.1. Distribution was by Biological Processes among those genes with associated GO terms according to the Blast2-GO software (level 3).

Utility of the EST collection from *F. xananassa *was finally tested in transcriptomic studies. One set of 6,349 *Fragaria xananassa *non redundant sequences here reported, and another set of 7,734 *Fragaria vesca *non redundant sequences available in the GenBank, were used to design an oligo-based microarray for the expression studies. Fruit characteristics of the cultivated *F. xananassa *are very different from the *F. vesca *in terms of size, colour, softness and volatiles [3]. These differences have their origin in the receptacle tissue and become more apparent at the ripe stage. Therefore, analysis of transcripts was performed in the receptacle of ripe fruits from cultivated strawberry *F. xananassa *(cv. Camarosa) and *F. vesca*. Expression values are provided in the Additional File 5. Prior to the analysis of the results, redundancy between the two sets of sequences was determined. A blastN between both datasets with an e-value < 1e-100, and a similarity percentage > 90% were used as discriminatory criteria. Global analysis was restricted to genes with very different expression level in the two species. Thus, Figure 3 shows the results of the genes that were over 4-fold up- (Figure 3A, 892 genes) and down-regulated (Figure 3B, 269 genes) in *F. xananassa *relative to *F. vesca*, analyzed by GO terms, when differences were statistically significant (p value ≤ 0.1). in general distribution of genes between categories of up- and down-regulated genes was similar between them, and also to the distribution in the *F. xananassa *EST collection here analyzed (Figure 1). However, there are two categories where differences, albeit minor, appear meaningful. The category "response to stress" was more abundant among the genes up-regulated in *F vesca *(13.1%) in comparison to those up-regulated in *F. xananassa *(4.46%). Most of these genes correspond to heat shock proteins (Table 10), which have been reported to play a role not only in thermo tolerance but also in plant development [47], [48]. A second difference was observed in the category of "regulation of cellular processes" that was more highly represented among the genes up-regulated in *F. xananassa*. Detailed analysis of the genes reveals that most of them encode for proteins involved in signalling processes, some of them related to hormone action, especially auxin (Table 11).

**Table 10:** Sequences down-regulated in the receptacle of red fruits of *Fragaria xananassa *(cv. Camarosa) in comparison to red receptacle of *F. vesca*, corresponding to the biological process "Response to Stress" using the Blat2go software.

**GenBank Acc. No**.	Fold change DOWN *F. xananassa*/*F. vesca*	Sequence description
DY674268	30.9	small heat-shock
GT151847	25.0	small heat shock protein
DY671878	18.4	heat shock protein 18
CO817068	16.9	thaumatin-like protein precursor
DY673748	14.0	hypothetical protein
DY668381	9.5	ripening-induced protein
DY667780	6.3	major latex
DV439443	6.0	heat shock protein 83
GT150079	5.8	heat shock
DY674688	5.2	heat shock
GT149690	4.8	heat shock protein
GT150402	4.3	heat shock protein putative
CX662201	4.2	mitochondrial heat shock 22 kd
GT149286	4.1	heat shock protein 17.8

Expression study was performed in a microarray system as described in the Methods. Sequences were selected after establishing a 4-fold threshold and passing a t-test and FDR (Benjamini-Hochberg) test (p-value < 0.1) for significance.

**Table 11:** Sequences up-regulated in the receptacle of red fruits of *Fragaria xananassa *(cv. Camarosa) in comparison to red receptacle of *F. vesca*, corresponding to the biological process "Regulation of Cellular Processes" according to the Blat2go software.

**GenBank Acc. No**.	Fold change *F. ananassa*/*F. vesca *UP	Sequence Description
GT149212	226.0	LRR serine-threonine protein kinase
DY667473	81.3	LRR serine-threonine protein kinase
GW402842	79.8	rac gtp binding protein arac7
DV439123	40.4	serine-threonine protein kinase
GT150368	33.6	receptor protein
DY673796	31.7	serine-threonine protein kinase
DV438802	26.4	phosphoinositide binding
GT149729	17.6	type-b response regulator
GT149402	14.4	auxin-repressed protein
CX661768	14.0	auxin influx carrier protein
GT151327	9.3	gasa4-like protein
CX662081	7.4	auxin-induced protein
DY676267	6.0	glutamate-gated kainate-type ion channel receptor subunit 5
DY672414	5.3	auxin influx transport protein
GT150907	5.1	brassinosteroid insensitive 1- associated receptor kinase 1
DV439901	4.7	gasa4-like protein
DY675461	4.5	conserved hypothetical protein
DY676028	4.3	auxin-repressed protein
DY671913	4.2	two-component system sensor histidine kinase response
DV438250	4.2	conserved hypothetical protein

Expression study was performed in a microarray system as described in the Methods. Sequences were selected after establishing a 4-fold threshold and passing a t-test and FDR (Benjamini-Hochberg) test (p-value < 0.1) for significance.

## Discussion

Sequencing information has produced important data that is being used to investigate both basic and applied aspects of plant growth and development. It is the first step towards a functional genomics, and a basic tool for molecular breeding. However, this information has been mainly generated either in model species or species with great impact in global food supply. Fruits of cultivated strawberry (*F. xananassa*) are appreciated both as fresh and as processed foods. However, there have been only limited genetic and genomic resources developed in this species due to its growing characteristics and the inherent difficulty of working with an octoploid. Despite this, genetic and genomic information is slowly appearing and recently the first genetic map has been reported [42]. In this work we analyzed more than 10,000 ESTs from *F. xananassa*, assembled in more than 7,000 unigenes. Half of these sequences proceeded from our own sequencing project; a second set of sequences was obtained from the GenBank dbEST Database.

Regarding the new sequences reported here it is worth emphasizing that they proceed from different fruit parts (achenes and receptacle), at different developmental stages (green and red fruit), and after hormone treatment (ethylene). In addition to the genetic characteristics, difficulties analyzing strawberry fruit growth and ripening arise from the fact that the commercial fruit is not a true fruit but includes an engrossed flower receptacle with the true fruits, the achenes, attached on its surface. Moreover, the development pattern of these two parts of the commercial fruit is not synchronous in that the achenes reach their mature stage much earlier than receptacle [49] Thus, the sequence information provided in this report specific for achenes and receptacle libraries is highly valuable. This is highlighted by the high number of ESTs encoding prunins in the achene library that is absent in the receptacle library. Similarly a large number of ESTs encoding metallothioneins were identified in the receptacle library with a low number of ESTs in the achene library. Prunins are known as the globulins of the genus Prunus, which comprise the main family of storage proteins synthesized in seeds during embryogenesis [50]. Metallothioneins belong to a family of cysteine-rich, low molecular weight proteins that have the capacity to bind metals through the thiol group of the cysteine residues, which represent nearly 30% of their amino acidic residues. These proteins have been shown to be involved in metal scavenging and detoxification [51], as well as in biotic and abiotic plant responses [52], [53]. Their high abundance in green receptacle suggests their important role in this organ.

The gene index analysis of the sequences reflected the genetic proximity of strawberry with other species of Rosaceae. Effectively, *Fragaria *sp. belongs to the Rosaceae family that includes apple, peach and apricot, and to the Rosaceae supertribe [54] that includes rose. The highest identity in the alignment was with *F. vesca*, from the same genus, followed by *Rosa hybrida *from the same supertribe, and *Prunus *and *Malus *from the same family. Previous studies on genomic resources of *Fragaria *and *Rosa *have also shown a high level of genetic proximity [54], [55]. There are more than 50.000 ESTs available from the diploid *F. vesca *[55] that has been proposed as a model plant for genomic studies. Recent studies have predicted approximately 200 Mb for its genome size [56] which might facilitate its complete sequencing. However, cultivated strawberry is an octoploid species with at least two genomes involved in its origin; one is thought to be an ancestor of *F. vesca *or *F. manchurica*, and the other an ancestor of *F. iinumae*, or potentially other species [42].

Overall comparison between the *F. vesca *and *F. xananassa *has revealed that only 32.42% of the diploid species had a corresponding putative orthologous gene in the octoploid. A possible explanation for this low value would be that the *F. vesca *derived subgenome is silenced in *F. xananassa*, as it has been previously described for specific genes [57], or even that the donor subgenome could be an ancestor or *F. vesca*. However, these hypothesis needs further studies since it could also be just a consequence of the different precedence of the EST sequences used in this comparison, mostly from plantlets in *F. vesca *and from fruits in *F. xananassa*. In any case, cultivated strawberry still represents a great potential source of alleles that might be important for selected traits, since in other species it has been shown that polyploidization is accompanied by changes in the gene expression, and accordingly in phenotypic variation [58].

In addition, the strawberry fruit produces some metabolites that are not found in other fruit models, such as tomato. These aspects make the ESTs information provided here valuable since it might eventually be used to probe for specific genes in other species, some of them closely related as some berries of the *Rubus *genus, like raspberry and blackberry that are classified in the same supertribe of Rosoideae as *Fragaria *[54].

SSRs derived from ESTs have been used as functional markers in the generation of maps and in breeding programs. In strawberry, we have previously used some of these markers to study genetic diversity within the species [59]. Based on the high level of identity found with corresponding genes of genetically close species, like those of the Rosaceae family, we foresee their transferability to these species, as other authors have shown [60], [61]. For this purpose, it is important to indicate that strawberry comparative map reveals a high level of co-linearity between diploid and octoploid *Fragaria *species [42]. For other species of the Rosaceae family this transferability deserves to be evaluated.

The function played by hormones in the development of strawberry fruits is still an unresolved question. Considered as a non-climacteric fruit, the main role has been attributed to the auxin synthesized in the achenes [62]. A search for genes involved in hormones response was performed. Auxin response factors (ARF) are transcription factors acting on the signalling pathway of this hormone [63]. We have unequivocally identified two of them in the strawberry ESTs Database, *FaARF1 *and *FaARF3*. For the *FaARF1*, the highest homology corresponds to a gene expressed in tomato [64], and to the Arabidopsis *ARF1 *gene [65]. *FaARF3 *has high homology to both *ARF3 *genes from tomato and Arabidopsis. The strawberry gene *FaARF3 *is mostly expressed in the receptacle at the white stage. At this stage the content of auxin is decreasing but still high in comparison to red fruits [8], [62], and cell expansion determines the final size of the receptacle.

The ethylene binding factors (ERF) constitute a family of transcription factors that were identified by their capacity to bind ethylene-responsive elements (ERE) present as *cis*-sequences in the ethylene-inducible genes. Further studies revealed that they act as transcriptional activators or repressors of GCC Box-mediated gene expression [44]. In tomato fruits it has been reported that some of them participate in the signalling pathway initiated by ethylene during the ripening of the fruits [32]. In the ESTs collection we have identified three putative ERFs (*FaERF1*, *FaERF2*, *FaERF3*) proceeding from the library prepared from the achenes, and this is consistent with the finding that achenes produced four to ten-fold more ethylene than fruit epidermal peels [5]. Both *FaERF1 *and *FaERF3 *have highest expression at the green stage and show high homology with *SlERF2 *[66] and *MdERF1 *[67], respectively, involved in tomato and apple fruit ripening. The corresponding Arabidopsis genes for *FaEFR1 *and *FaERF3 *belong to the subfamily B-2 (Group VII) [68]. In contrast, the Arabidopsis gene homologous to *FaERF2*, which shows minor variation, is classified in the subfamily B-3 (Group IX) [68]. The genes in group IX have often been linked in defensive gene expression in response to pathogen infection.

In strawberry there is no information on the content of active brassinosteroid in the ripening fruit. The preferential expression of *FaBRI1 *in red receptacle suggests an increased concentration of this hormone in this tissue at later stages of ripening. However, a relationship between *FaBRI1 *expression and an increased concentration is not direct since it is needed to know the expression of other important elements in the brassinosteroid signalling pathway such as BAK1 (BRI1 associated receptor kinase) and BKI1 (inhibitor of the association of BRI1 and BAK1) [69]. BZR1 is a transcription factor [70] whose cell location depends on its phosphorylation status, mainly controlled by BIN2 [71]. When BZR1 is phosphorylated goes to the nucleus where induces the expression of brassinosteroid dependent genes. Expression of genes *FaBZR *and *FaBIN2 *occurs in achenes and receptacle at all stages, but the expression ratio *FaBZR/FaBIN2 *is higher in the white achene and lower in white receptacle. These expression patterns must be interpreted under the light of the interaction of the encoded proteins as above indicated. In summary, the functional relevance of all these expression studies in terms of the role of hormones in fruit ripening is limited. However, they illustrate the possibility of using the sequence information here reported to initiate the molecular dissection the problem with gene-specific tools.

The database here reported allowed the comparison of the transcriptome in the ripe receptacle of *F. xananassa *(cv. Camarosa) and the diploid *F. vesca*. As expected, there are very specific changes in genes related to secondary metabolism (see Additional file 6). However, global analysis revealed that differences in the transcriptomes being more quantitative than qualitative i.e. supported by activation/depletion rather than by gain/loss of biological processes. The two minor differences found in "response to stress", up-regulated in *F. vesca*, and "regulation of cellular processes", up-regulated in *F*. xananassa, are probably related to the domestication of the species. Natural environment of the wild *F. vesca *is more cold climate and high altitude than *F. xananassa *[3], and it is probable that its cultivation under temperate conditions triggers the heat stress response. On the other hand, is not surprising that hormone signalling pathways are more efficient in *F. xananassa *especially those related to auxin action since it has been reported that increasing auxin content in both *F. xananassa *and *F. vesca *has the effect of increasing weight and size of fruits [72]. The relevance of these changes here reported deserves further investigation by a deep study of specific genes. This is currently under progress.

## Conclusions

We anticipate that the generation of this strawberry gene dataset will be important in further genomic studies of this species. It doubled the number of ESTs available for this species and combined and analysed all the information presently available for the strawberry. The analysis of the information reported and gathered in relation to the cultivated strawberry when compared with the available information on the wild strawberry, the diploid *Fragaria vesca*, is valuable to establish their genetic relationship. It is an essential source of information for the study of the expression of genes, either by QRT-PCR or by microarray. It will also allow the establishment of few tools for the analysis of metabolic and hormone signalling pathways playing a role in the different developmental processes of this species.

## Methods

### Plant material

Strawberry plants (*F. xananassa *Duchesne ex. Rozier) were grown under field conditions in Huelva, in the southwest of Spain. The fruits were sampled at selected developmental stages that we previously established [10]. For the expression studies samples were from receptacle and achenes, separately, from stages of green fruit (green receptacle and green achenes); white fruits: white receptacle and green achenes; and red fruits: red receptacle and brown achenes, of the cultivar Camarosa. The cDNA libraries were prepared from different tissues of the strawberry fruits at various developmental stages. The M1 and M2 libraries were prepared from receptacle and achenes, respectively, of fruits of the cultivar Carisma at the green stage. The C1, and C2, and C3 libraries were prepared from fruits of the cultivar Chandler, being C1 and C3 subtractive libraries. Whereas libraries C1 and C2 were prepared from whole fruits, the C2 library was only from receptacle. The L1 library was prepared from red fruits (receptacle and achenes) of the cultivar Elsanta treated with ethylene.

In the microarray studies, plants of *F. xananassa *(cv. Camarosa) and F. *vesca *were cultivated in a greenhouse under natural light conditions in Churriana (Málaga, Spain), and fruits of the two species were sampled during their overlapping growing season.

### Construction of cDNA libraries and EST sequencing

For the M1 and M2 libraries achenes were removed from fruits at the green stage and total RNA was extracted separately from the remaining receptacle and the achenes. Total RNA isolation was performed as previously described [73]. Poly(A+) mRNA was purified from total RNA using the 'PolyAtract_mRNA Isolation Systems' kit according to the manufacturer's instruction (Promega). This poly(A+) RNA was used for the construction of the directional cDNA library in the Lambda ZAP Express phage using the 'ZAP Express_ cDNA Synthesis Kit','Gigapack_ III Gold Cloning Kit', and 'Gigapack_ III GoldPackaging Extract' kits according to the manufacturer's instructions (Stratagene, La Jolla, CA).

The C1 subtractive library (red stage versus green stage) was generated from the whole fruit (receptacle and achenes) as previously described [74]. The C2 library was prepared from RNA extracted from whole red strawberry fruits [16]. The C3 library was prepared based in the suppression subtractive hybridization (SSH) [75]. The subtraction (red stage versus green stage) was normalized and prepared, only from receptacle tissue, according the Clontech PCR-Select cDNA Subtraction Kit (BD Biosciences) system. For the L1 library ripe strawberry fruits were exposed to a constant stream of air containing 50 vpm ethylene. RNA was extracted after 2, 4, 24, 48 and 72 hours and also used in a suppressed subtractive hybridisation (SSH, Clontech Inc.) protocol.

Sequencing of the M1 and M2 libraries was performed from the 5'-end of the inserts using the M13 reverse primer by a custom service (Sistemas Genómicos S.L., Spain). The C1, C2, and C3 libraries were sequenced in an ABI PRISM™ 310 de Perkin Elmer by the Central Services of the Universidad de Córdoba. Primers used were T3, T7, M13 forward y M13 reverse when cloned in pBSII, and sp6 when cloned in pGEM-T.

### Bioinformatics

The strawberry EST sequences for the libraries CO3, CO8 and SGBL were obtained from the dbEST database from GenBank. Libraries with less than 100 sequences were placed in the group SGBL (Small GenBank libraries).

EST sequences were cleaned with the seqclean software [41] using the default parameters. As dataset for fragments of vectors and adaptors the Univec and Univec_core from NCBI were used. To remove contaminants, ColiBank95, an Escherichia coli genome dataset from NCBI, was used. The program was repetitively applied to the sequences in FASTA format until no sequence was excluded. Clustering and creation of the consensus sequences were performed through the TGICL pipeline [41] with the programs Megablast for clustering and Cap3 for the consensus sequences. Variations on the default parameters in Megablast revealed that the percentage of minimum identity was the only determinant on the final number of clusters. Thus, the parameters established for clustering were: 95 percent for the minimum identity, 40 bp length for the minimum overlapping region, and 20 bp length for maximum non-overlapping extremes. Those sequences from a cluster allowing the establishment of a consensus sequence were included in a contig. In this process, we defined singlets as clustered sequences that could not be included in a consensus sequence and singletons as sequences that were not grouped in a cluster. The unigenes were then the sum of singletons, singlets, and contigs.

The chimera analysis was performed parsing the results of the BlastX of the 5' and 3' extremes (300 nt) of each unigene using TAIR 8 as blast database. Unigenes that presented different blast hits for each extreme not related between them were annotated as putative chimeras.

Functional annotation was performed using the package Blast2Go [40]. Tools of this package were used for BlastX (using GenBank nr as database and 1e-10 as initial cutoff e-value), InterProScan (for protein domain search and signal peptide prediction) and enzyme code and GO term mapping. The functional category analysis was done over biological process GO term distribution at a cutoff level of 3.

The datasets for the comparison with other species were made in the following way: The sequences were downloaded from the dbEST database in GenBank. These sequences were cleaned and clustered in the same way as the strawberry sequences. The homology search between strawberry and these species were made with the program Blastall, and subprogram TblastX, with a threshold e-value of 1e-20.

### SSRs and SNPs

The identification and localization of SSRs was accomplished by PERL5 scripts MISA [76]. SSRs were only considered when they contained motifs that were between two and five nucleotides in size and with 2, 3, 4 and 5 repeats for di- tri-tetra- and pentanucleotides, respectively. For SNP location we have used the pipeline QualitySNP [77] that develops an algorithm to detect reliable SNPs and insertions/deletions in EST data, from diploid and polyploid species. The default parameters were used, i.e. CAP3 similarity of overlap 95%, minimum size of alleles of each SNP 2, length of the low quality region at the 5' end of sequence 30 nucleotides, similarity on one polymorphic site 0.75, similarity on all polymorphic ie sites 0.8, low quality region of 3' side 0.2 (20% of the whole sequence). The weight value of the low quality region 0.5 and the minimal confidence score 2. 2.

### Expression studies

Total RNA was extracted from *F. xananassa *fruits, from receptacle and achenes separatetly, according to the method described by [74]. Two biological and three technical replicates of each were performed for every sample. The RT reaction was done using iScript ™cDNA Synthesis Kit (Bio-Rad, http://www.bio-rad.com) according to the manufacturer's instructions. Expression was analysed by real-time quantitative RT-PCR using iQ™SYBR^® ^Green Supermix sample in an iCycler detection system (Bio-Rad, http://www.bio-rad.com according to the manufacturer's instructions, and gene-specific primers. The results obtained were normalized against FaRIB413 expression that was reported to be constitutive [78]. The primers used in the PCR reactions are indicated in Additional File 6.

### Microarray analysis

Oligo (60 mer length) design for expression analysis was performed by NimbleGen Systems Inc. from 6,349 non redundant sequences of *F. xananassa *http://fresa.uco.uma.es/srs71 and 7,734 non redundant sequences of *F. vesca *(GenBank). A minimum of 7-10 oligo were printed per probe and three blocks were printed per dataset. Samples corresponding to two growing seasons were prepared as high quality double-stranded cDNA which were synthesized from total RNA, extracted from the receptacle of ripe fruit as above described, following the protocol described in the Invitrogen's SuperScript™ Double-Stranded cDNA Synthesis Kit. Samples labeling, hybridization with three probes per target, and data normalization was performed by NimbleGen Systems Inc. according to the procedures described in the expression analysis section http://www.nimblegen.com/.

Data analysis of the microarrays expression studies was performed with the software for gene expression analysis ArrayStar (DNASTAR). The t-test and FDR (Benjamini-Hochberg) for multiple testing corrections were used with a confidence p-value < 0.1, to identify statistically significant differences.

The redundancy between probes of the two species were analysed using BlastN with cutoff value < 1e-100 and a similarity percentage > 90%.

## Authors' contributions

AB: preparation of the libraries, EST sequencing quality assurance, data analysis, cloning and characterization the brassinosteroid-related genes, expression studies by microarrays, preparation of the manuscript;

CM: cloning and characterization the ethylene-related genes, preparation of the manuscript;

FC: cloning and characterization the auxin-related genes, preparation of the manuscript;

EC-R: expression studies by QRT-PCR and microarrays;

JLC: preparation of the libraries, sequencing, data analysis

NM-E: expression studies by microarrays, preparation of the manuscript

RB-P: sequencing, data analysis

MAB: data analysis, preparation of the manuscript

JM-B: preparation of the libraries, sequencing, data analysis

JFSS: sequence processing, assembly and annotation, database development, preparation of the manuscript.

VV: overall project co-ordination and supervision, preparation of the manuscript

## Supplementary Material

Additional file 1 **Representative unigenes of the biosynthetic pathways of sugars, lipids, amino acids and nucleotides**. Number of ESTs identified per library of Table 1 classified as singletons and contigs, which correspond to genes of the biosynthetic pathways of sugars, lipids, amino acids and nucleotides, and values of their relative abundance in libraries M1 and M2. Click here for file

Additional file 2 **Representative unigenes of the flavonoids and hormones biosynthetic pathways**. Number of ESTs identified per library of Table 1 classified as singletons and contigs, which correspond to genes of the biosynthetic pathways of flavonoids and hormones, and values of their relative abundance in libraries. Click here for file

Additional file 3 **Representative unigenes involved in ethylene signaling**. Number of ESTs identified per library of Table 1 classified as singletons and contigs, which correspond to genes of the ethylene signalling pathway, and values of their relative abundance in libraries M1 and M2. Click here for file

Additional file 4 **Representative unigenes involved in cell wall biochemistry**. Number of ESTs identified per library of Table 1 classified as singletons and contigs, which correspond to genes involved in cell wall biochemistry, and values of their relative abundance in libraries M1 and M2. Click here for file

Additional file 5 **Expression results in *F. xananassa *and *F. vesca *ripe receptacle performed by a microarray designed from EST sequences of this two species**. Mean values of the hybridization signals of the probes, identified by the GenBank Acc. No. and the GO terms, used in the microarray expression study. The file include values of the comparison between *F. xananassa *and F. vesca, including the P and T values. Click here for file

Additional file 6 **Sequences of the primers used in the expression studies by QRT-PCR**. Nucleotide sequence of the primers used for the expression studies of the genes of the hormones signalling pathways of Table 9 Click here for file

## References

[B1] FAOSTAT http://faostat.fao.org/site/567/DesktopDefault.aspx

[B2] Seeram NP Berry Fruits: Compositional Elements, Biochemical Activities, and the Impact of Their Intake on Human Health, Performance, and Disease J Agric Food Chem 2008 56 627 62 10.1021/jf071988k 18211023

[B3] Hancock JF Strawberries 2000 CABI

[B4] Trainotti L Pavanello A Casadoro G Different ethylene receptors show an increased expression during the ripening of strawberries: does such an increment imply a role for ethylene in the ripening of these non-climacteric fruits? J Exp Bot 2005 56 2037 2046 10.1093/jxb/eri202 15955790

[B5] Iannetta PPM Laarhoven L Medina-Escobar N James EK McManus MT Davies HV Harren FJM Ethylene and carbon dioxide production by developing strawberries show a correlative pattern that is indicative of ripening climacteric fruit Physiol Plantarum 2006 127 247 259 10.1111/j.1399-3054.2006.00656.x

[B6] Nitsch JP Free Auxins and Free Tryptophane in the Strawberry Plant Physiol 1995 30 33 39 10.1104/pp.30.1.33 PMC540593 16654724

[B7] Given NK Venis MA Gierson D Hormonal regulation of ripening in the strawberry, a non-climacteric fruit Planta 1988 174 402 406 10.1007/BF00959527 24221523

[B8] Nitsch JP Growth and Morphogenesis of the Strawberry as Related to Auxin Am J Bot 1950 57 211 215 10.2307/2437903

[B9] Halbwirth H Puhl I Haas U Jezik K Treutter D Stich K Two-Phase Flavonoid Formation in Developing Strawberry (*Fragaria xananassa*) Fruit J Agric Food Chem 2006 54 1479 1485 10.1021/jf0524170 16478277

[B10] Agius F Gonzalez-Lamothe R Caballero JL Muñoz-Blanco J Botella MA Valpuesta V Engineering increased vitamin C levels in plants by overexpression of a D-galacturonic acid reductase Nat Biotechnol 2003 21 177 181 10.1038/nbt777 12524550

[B11] Aharoni A Keizer LPC Bouwmeester HJ Sun Z Alvarez-Huerta M Verhoeven HA Blaas J van Houwelingen AMML De Vos RCH van der Voet H Jansen RC Guis M Mol J Davis RW Schena M van Tunen AJ O'Connell AP Identification of the SAAT Gene Involved in Strawberry Flavor Biogenesis by Use of DNA Microarrays Plant Cell 2000 12 647 662 10.1105/tpc.12.5.647 10810141 PMC139918

[B12] Roscher R Koch H Herderich M Schreier P Schwab W Identification of 2,5-dimethyl-4-hydroxy-3[2H]-furanone beta-D-glucuronide as the major metabolite of a strawberry flavour constituent in humans Food Chem Toxicol 1997 35 777 782 10.1016/S0278-6915(97)00055-0 9350222

[B13] Raab T Lopez-Raez JA Klein D Caballero JL Moyano E Schwab W Muñoz-Blanco J FaQR, Required for the Biosynthesis of the Strawberry Flavor Compound 4-Hydroxy-2,5-Dimethyl-3(2H)-Furanone, Encodes an Enone Oxidoreductase Plant Cell 2006 18 1023 1037 10.1105/tpc.105.039784 16517758 PMC1425863

[B14] Lunkenbein S Salentijn EMJ Coiner HA Boone MJ Krens FA Schwab W Up- and down-regulation of *Fragaria xananassa *O-methyltransferase: impacts on furanone and phenylpropanoid metabolism J Exp Bot 2006 57 2445 2453 10.1093/jxb/erl008 16798852

[B15] Civello PM Powell AL Sabehat A Bennett AB An Expansin Gene Expressed in Ripening Strawberry Fruit Plant Physiol 1999 121 1273 1279 10.1104/pp.121.4.1273 10594114 PMC59494

[B16] Trainotti L Spolaore S Pavanello A Baldan B Casadoro G A novel E-type endo-beta-1,4-glucanase with a putative cellulose-binding domain is highly expressed in ripening strawberry fruits Plant Mol Biol 1999 40 323 332 10.1023/A:1006299821980 10412910

[B17] Trainotti L Spinello R Piovan A Spolaore S Casadoro G β-Galactosidases with a lectin-like domain are expressed in strawberry J Exp Bot 2001 52 1635 1645 10.1093/jexbot/52.361.1635 11479328

[B18] Medina-Escobar N Cárdenas J Moyano E Caballero JL Muñoz-Blanco J Cloning, molecular characterization and expression pattern of a strawberry ripening-specific cDNA with sequence homology to pectate lyase from higher plants Plant Mol Biol 1997 34 867 877 10.1023/A:1005847326319 9290639

[B19] Benitez-Burraco A Blanco-Portales R Redondo-Nevado J Bellido ML Moyano E Caballero J Munoz-Blanco J Cloning and characterization of two ripening-related strawberry (*Fragaria xananassa *cv. Chandler) pectate lyase genes J Exp Bot 2003 54 633 645 10.1093/jxb/erg065 12554706

[B20] Castillejo C de la Fuente JI Iannetta P Botella MA Valpuesta V Pectin esterase gene family in strawberry fruit: study of FaPE1, a ripening-specific isoform J Exp Bot 2004 55 909 918 10.1093/jxb/erh102 15020638

[B21] Osorio S Castillejo C Quesada MA Medina-Escobar N Brownsey GJ Suau R Heredia A Botella MA Valpuesta V Partial demethylation of oligogalacturonides by pectin methyl esterase1 is required for eliciting defence responses in wild strawberry (*Fragaria vesca*) Plant J 2008 54 43 55 10.1111/j.1365-313X.2007.03398.x 18088306

[B22] Fait A Hanhineva K Beleggia R Dai N Rogachev I. Nikiforova VJ Fernie AR Aharoni A Reconfiguration of the achene and receptacle metabolic networks during strawberry fruit development Plant Physiol 2008 148 730 750 10.1104/pp.108.120691 18715960 PMC2556830

[B23] Folta KM Staton M Stewart PJ Jung S Bies DH Jesdurai C Main D Expressed sequence tags (ESTs) and simple sequence repeat (SSR) markers from octoploid strawberry (*Fragaria xananassa*) BMC Plant Biology 2005 5 12 10.1186/1471-2229-5-12 15985176 PMC1182381

[B24] Gil-Ariza D Amaya I Botella MA Muñoz-Blanco J Caballero JL López Aranda J Valpuesta V Sánchez-Sevilla J EST-derived polymorphic microsatellites from cultivated strawberry (*Fragaria xananassa*) are useful for diversity studies and varietal identification among *Fragaria *species Mol Ecol Notes 2006 6 1195 1197 10.1111/j.1471-8286.2006.01489.x

[B25] Aharoni A O'Connell AP EST-derived polymorphic microsatellites from cultivated strawberry (*Fragaria xananassa*) are useful for diversity studies and varietal identification among *Fragaria *species J Exp Bot 2002 53 2073 2087 10.1093/jxb/erf026 12324531

[B26] Aharoni A Keizer LC Van Den Broeck HC Blanco-Portales R Munoz-Blanco J Bois G Smit P De Vos RC O'Connell AP Novel Insight into Vascular, Stress, and Auxin-Dependent and -Independent Gene Expression Programs in Strawberry, a Non-Climacteric Fruit Plant Physiol 2002 129 1019 1031 10.1104/pp.003558 12114557 PMC166497

[B27] Salentijn EMJ Aharoni A Schaart JG Boone MJ Krens FA Differential gene expression analysis of strawberry cultivars that differ in fruit-firmness Physiol Plantarum 2003 118 571 578 10.1034/j.1399-3054.2003.00138.x

[B28] Ewing RM Ben Kahla A Poirot O Lopez F Audic S Claverie JM Large-scale statistical analyses of rice ESTs reveal correlated patterns of gene expression Genome Res 1999 9 950 959 10.1101/gr.9.10.950 10523523 PMC310820

[B29] Sargent DJ Clarke J Simpson DW Tobutt KE Arús P Monfort A Vilanova S Denoyes-Rothan B Rousseau M Folta KM Bassil NV Battey NH An enhanced microsatellite map of diploid *Fragaria* Theor Appl Genet 2006 112 1349 1359 10.1007/s00122-006-0237-y 16505996

[B30] Choi I Hyten DL Matukumalli LK Song Q Chaky JM Quigley CV Chase K Lark KG Reiter RS Yoon M Hwang E Yi S Young ND Shoemaker RC van Tassell CP Specht JE Cregan PB A Soybean Transcript Map: Gene Distribution, Haplotype and Single-Nucleotide Polymorphism Analysis Genetics 2007 176 685 696 10.1534/genetics.107.070821 17339218 PMC1893076

[B31] Zhang BH Pan XP Wang QL Cobb GP Anderson TA Identification and characterization of new plant microRNAs using EST analysis Cell Res 2005 15 336 360 10.1038/sj.cr.7290302 15916721

[B32] Alba R Payton P Fei Z McQuinn R Debbie P Martin GB Tanksley SD Giovannoni JJ Transcriptome and Selected Metabolite Analyses Reveal Multiple Points of Ethylene Control during Tomato Fruit Development Plant Cell 2005 17 2954 2965 10.1105/tpc.105.036053 16243903 PMC1276022

[B33] Waters DL Holton TA Ablett EM Lee LS Henry RJ The ripening wine grape berry skin transcriptome Plant Science 2006 171 132 138 10.1016/j.plantsci.2006.03.002

[B34] Fei Z Tang X Alba RM White JA Ronning CM Martin GB Tanksley SD Giovannoni JJ Comprehensive EST analysis of tomato and comparative genomics of fruit ripening Plant J 2004 40 47 59 10.1111/j.1365-313X.2004.02188.x 15361140

[B35] Goes da Silva F Iandolino A Al-Kayal F Bohlmann MC Cushman MA Lim H Ergul A Figueroa R Kabuloglu EK Osborne C Rowe J Tattersall E Leslie A Xu J Baek J Cramer GR Cushman JC Cook DR Characterizing the Grape Transcriptome. Analysis of Expressed Sequence Tags from Multiple Vitis Species and Development of a Compendium of Gene Expression during Berry Development Plant Physiol 2005 139 574 597 10.1104/pp.105.065748 16219919 PMC1255978

[B36] Newcomb RD Crowhurst RN Gleave AP Rikkerink EH Allan AC Beuning LL Bowen JH Gera E Jamieson KR Janssen BJ Laing WA McArtney S Nain B Ross GS Snowden KC Souleyre EJ Walton EF Yauk Y Analyses of Expressed Sequence Tags from Apple Plant Physiol 2006 141 147 166 10.1104/pp.105.076208 16531485 PMC1459330

[B37] Forment J Gadea J Huerta L Abizanda L Agusti J Alamar S Alos E Andres F Arribas R Beltran JP Berbel A Blazquez MA Brumos J Canas LA Cercos M Colmenero-Flores JM Conesa A Estables B Gandia M Garcia-Martinez JL Gimeno J Gisbert A Gómez G Gonzalez-Candelas L Granell A Guerri J Lafuente MT Madueno F Marcos JF Marques MC Martinez F Martinez-Godoy MA Miralles S Moreno P Navarro L Pallas V Perez-Amador MA Perez-Valle J Pons C Rodrigo I Rodriguez PL Royo C Serrano R Soler G Tadeo F Talon M Terol J Trenor M Vaello L Vicente O Vidal C Zacarias L Conejero V Development of a citrus genome-wide EST collection and cDNA microarray as resources for genomic studies Plant Mol Biol 2005 57 375 391 10.1007/s11103-004-7926-1 15830128

[B38] Gonzalez-Ibeas D Blanca J Roig C Gonzalez-To M Pico B Truniger V Gómez P Deleu W Cano-Delgado A Arus P Nuez F Garcia-Mas J Puigdomenech P Aranda M MELOGEN: an EST database for melon functional genomics BMC Genomics 2007 8 306 10.1186/1471-2164-8-306 17767721 PMC2034596

[B39] Quackenbush J Liang F Holt I Pertea G Upton J The TIGR gene indices: reconstruction and representation of expressed gene sequences Nucleic Acids Res 2000 28 141 145 10.1093/nar/28.1.141 10592205 PMC102391

[B40] Conesa A Götz S García-Gómez JM Terol J Talón M Robles M Blast2GO: a universal tool for annotation, visualization and analysis in functional genomics research Bioinformatics 2005 21 3674 3676 10.1093/bioinformatics/bti610 16081474

[B41] Pertea G TIGR Gene Indices clustering tools (TGICL): a software system for fast clustering of large EST datasets Bioinformatics 2003 19 651 10.1093/bioinformatics/btg034 12651724

[B42] Rousseau-Gueutin M Lerceteau-Kohler E Barrot L Sargent DJ Monfort A Simpson D Arus P Guerin G Denoyes-Rothan B Comparative Genetic Mapping Between Octoploid and Diploid Fragaria Species Reveals a High Level of Colinearity Between Their Genomes and the Essentially Disomic Behavior of the Cultivated Octoploid Strawberry Genetics 2008 179 2045 2060 10.1534/genetics.107.083840 18660542 PMC2516079

[B43] Jones B Frasse P Olmos E Zegzouti H Li ZG Latch A Pech JC Bouzayen M Down-regulation of DR12, an auxin-response-factor homolog, in the tomato results in a pleiotropic phenotype including dark green and blotchy ripening fruit Plant J 2002 32 603 613 10.1046/j.1365-313X.2002.01450.x 12445130

[B44] Fujimoto SY Ohta M Usui A Shinshi H Ohme-Takagi M Arabidopsis Ethylene-Responsive Element Binding Factors Act as Transcriptional Activators or Repressors of GCC Box-Mediated Gene Expression Plant Cell 2000 12 393 404 10.1105/tpc.12.3.393 10715325 PMC139839

[B45] Vardhini BV Rao SSR Acceleration of ripening of tomato pericarp discs by brassinosteroids Phytochemistry 2002 61 843 847 10.1016/S0031-9422(02)00223-6 12453577

[B46] Symons GM Davies C Shavrukov Y Dry IB Reid JB Thomas MR Grapes on Steroids. Brassinosteroids Are Involved in Grape Berry Ripening Plant Physiol 2006 140 150 158 10.1104/pp.105.070706 16361521 PMC1326039

[B47] Medina-Escobar N Cárdenas J Muñoz-Blanco J Caballero JL Cloning and molecular characterization of a strawberry fruit ripening-related cDNA corresponding a mRNA for a low-molecular-weight heat-shock protein Plant Mol Biol 1998 36 33 42 10.1023/A:1005994800671 9484460

[B48] Mittal D Chakrabarti S Sarkar A Singh A Grover A Heat shock factor gene family in rice: genomic organization and transcript expression profiling in response to high temperature, low temperature and oxidative stresses Plant Physiol Biochem 2009 47 785 795 10.1016/j.plaphy.2009.05.003 19539489

[B49] Perkins Veazie P Growth and ripening of strawberry fruit Horticultural Reviews 1995 17 267 197

[B50] Garcia-Mas J Messeguer R Arús P Puigdomenech P Molecular characterization of cDNAs corresponding to genes expressed during almond (Prunus amygdalus Batsch) seed development Plant Mol Biol 1995 27 205 210 10.1007/BF00019192 7865791

[B51] Hall J Cellular mechanisms for heavy metal detoxification and tolerance J Exp Bot 2002 53 1 11 10.1093/jexbot/53.366.1 11741035

[B52] Butt A Mousley C Morris K Beynon J Can C Holub E Greenberg JT Buchanan-Wollaston V Differential expression of a senescence-enhanced metallothionein gene in Arabidopsis in response to isolates of Peronospora parasitica and Pseudomonas syringae Plant J 1998 16 209 221 10.1046/j.1365-313x.1998.00286.x 9839466

[B53] Clement M Lambert A Herouart D Boncompagni E Identification of new up-regulated genes under drought stress in soybean nodules Gene 2008 426 15 22 10.1016/j.gene.2008.08.016 18817859

[B54] Potter D Eriksson T Evans RC Oh S Smedmark JEE Morgan DR Kerr M Robertson KR Arsenault M Dickinson TA Campbell CS Phylogeny and classification of Rosaceae Plant Syst Evol 2007 266 5 43 10.1007/s00606-007-0539-9

[B55] Shulaev V Korban SS Sosinski B Abbott AG Aldwinckle HS Folta KM Iezzoni A Main D Arus P Dandekar AM Lewers K Brown SK Davis TM Gardiner SE Potter D Veilleux RE Multiple Models for Rosaceae Genomics Plant Physiol 2008 147 985 1003 10.1104/pp.107.115618 18487361 PMC2442536

[B56] Pontaroli AC Rogers RL Zhang Q Shields ME Davis TM Folta KM SanMiguel P Bennetzen JL Gene Content and Distribution in the Nuclear Genome of *Fragaria vesca* Plant Genome 2009 2 93 101 10.3835/plantgenome2008.09.0007

[B57] Aharoni A Giri AP Verstappen FWA Bertea CM Sevenier R Sun Z Jongsma MA Schwab W Bouwmeester HJ Gain and Loss of Fruit Flavor Compounds Produced by Wild and Cultivated Strawberry Species Plant Cell 2004 16 3110 3131 10.1105/tpc.104.023895 15522848 PMC527202

[B58] Adams KL Cronn R Percifield R Wendel JF Genes duplicated by polyploidy show unequal contributions to the transcriptome and organ-specific reciprocal silencing Proc Natl Acad Sci USA 2003 100 4649 4654 10.1073/pnas.0630618100 12665616 PMC153610

[B59] Gil-Ariza D Amaya I López-Aranda JM Sanchez-Sevilla JF Botella MA Valpuesta V Impact of Plant Breeding on the Genetic Diversity of Cultivated Strawberry as Revealed by Expressed Sequence Tag-derived Simple Sequence Repeat Markers J Amer Soc Hort Sci 2009 134 337 347

[B60] Davis TM DiMeglio LM Yang R Styan SM Lewers KS Assessment of SSR Marker Transfer from the Cultivated Strawberry to Diploid Strawberry Species: Functionality, Linkage Group Assignment, and Use in Diversity Analysis J Amer Soc Hort Sci 2006 131 506 512

[B61] Monfort A Vilanova S Davis TM Arús P A new set of polymorphic simple sequence repeat (SSR) markers from a wild strawberry (*Fragaria vesca*) are transferable to other diploid Fragaria species and to *Fragaria xananassa* Mol Ecol Notes 2006 6 197 200 10.1111/j.1471-8286.2005.01191.x

[B62] Benjamins R Scheres B Auxin: the looping star in plant development Annu Rev Plant Biol 2008 59 443 465 10.1146/annurev.arplant.58.032806.103805 18444904

[B63] Tiwari SB Hagen G Guilfoyle T The roles of auxin response factor domains in auxin-responsive transcription Plant Cell 2003 15 533 543 10.1105/tpc.008417 12566590 PMC141219

[B64] Serrani JC Ruiz-Rivero O Fos M García-Martínez JL Auxin-induced fruit-set in tomato is mediated in part by gibberellins Plant J 2008 56 922 934 10.1111/j.1365-313X.2008.03654.x 18702668

[B65] Ellis CM Nagpal P Young JC Hagen G Guilfoyle TJ Reed JW AUXIN RESPONSE FACTOR1 and AUXIN RESPONSE FACTOR2 regulate senescence and floral organ abscission in Arabidopsis thaliana Development 2005 132 4563 4574 10.1242/dev.02012 16176952

[B66] Pirrello J Jaimes-Miranda F Sanchez-Ballesta MT Tournier B Khalil-Ahmad Q Regad F Latche A Pech JC Bouzayen M Sl-ERF2, a Tomato Ethylene Response Factor Involved in Ethylene Response and Seed Germination Plant Cell Physiol 2006 47 1195 1205 10.1093/pcp/pcj084 16857696

[B67] Wang A Tan D Takahashi A Zhong Li T Harada T MdERFs, two ethylene-response factors involved in apple fruit ripening J Exp Bot 2007 58 3743 3748 10.1093/jxb/erm224 18057044

[B68] Nakano T Suzuki K Fujimura T Shinshi H Genome-Wide Analysis of the ERF Gene Family in Arabidopsis and Rice Plant Physiol 2006 140 411 432 10.1104/pp.105.073783 16407444 PMC1361313

[B69] Wang X Chory J Brassinosteroids Regulate Dissociation of BKI1, a Negative Regulator of BRI1 Signaling, from the Plasma Membrane Science 2006 313 1118 1122 10.1126/science.1127593 16857903

[B70] Li L Den XW It runs in the family: regulation of brassinosteroid signaling by the BZR1-BES1 class of transcription factors Trends Plant Sci 2005 10 266 268 10.1016/j.tplants.2005.04.002 15949759

[B71] He J Gendron JM Yang Y Li J Wang Z The GSK3-like kinase BIN2 phosphorylates and destabilizes BZR1, a positive regulator of the brassinosteroid signaling pathway in Arabidopsis Proc Natl Acad Sci USA 2002 99 10185 10190 10.1073/pnas.152342599 12114546 PMC126645

[B72] Mezzetti B Landi L Pandolfini T Spena A The defH9-iaaM auxin- synthesizing gene increases plant fecundity and fruit production in strawberry and raspberry BMC Biotechnology 2004 4 4 10.1186/1472-6750-4-4 15113427 PMC394336

[B73] Manning K Isolation of nucleic acids from plants by differential solvent precipitation Anal Biochem 1991 195 45 50 10.1016/0003-2697(91)90292-2 1716071

[B74] Medina-Escobar N Cárdenas J Valpuesta V Muñoz-Blanco J Caballero JL Cloning and Characterization of cDNAs from Genes Differentially Expressed during the Strawberry Fruit Ripening Process by a MAST-PCR-SBDS Method Anal Biochem 1997 248 288 296 10.1006/abio.1997.2110 9177756

[B75] Diatchenko L Lau YF Campbell AP Chenchik A Moqadam F Huang B Lukyanov S Lukyanov K Gurskaya N Sverdlov ED Siebert PD Suppression subtractive hybridization: a method for generating differentially regulated or tissue-specific cDNA probes and libraries Proc Natl Acad Sci USA 1996 93 6025 6030 10.1073/pnas.93.12.6025 8650213 PMC39182

[B76] Thiel T Michalek W Varshney RK Graner A Exploiting EST databases for the development and characterization of gene-derived SSR-markers in barley (Hordeum vulgare L.) Theor Appl Genet 2003 106 411 22 12589540 10.1007/s00122-002-1031-0

[B77] Tang J Vosman B Voorrips R van der Linden CG Leunissen J QualitySNP: a pipeline for detecting single nucleotide polymorphisms and insertions/deletions in EST data from diploid and polyploid species BMC Bioinformatics 2006 7 438 10.1186/1471-2105-7-438 17029635 PMC1618865

[B78] Casado-Díaz A Encinas-Villarejo S Santos BLD Schilirò E Yubero-Serrano E Amil-Ruíz F Pocovi MI Pliego-Alfaro F Dorado G Rey M Romero F Muñoz-Blanco J Caballero J Analysis of strawberry genes differentially expressed in response to Colletotrichum infection Physiol Plantarum 2006 128 633 650 10.1111/j.1399-3054.2006.00798.x

[B79] de la Fuente JI Amaya I Castillejo C Sánchez-Sevilla JF Quesada MA Botella MA Valpuesta V The strawberry gene FaGAST affects plant growth through inhibition of cell elongation J Exp Bot 2006 57 2401 2411 10.1093/jxb/erj213 16804055

